# Molecular imaging along the heart-kidney axis

**DOI:** 10.7150/thno.102552

**Published:** 2024-10-21

**Authors:** Konrad Klimek, Daniel Groener, Xinyu Chen, Steven P. Rowe, Thimoteus Speer, Takahiro Higuchi, Rudolf A. Werner

**Affiliations:** 1 Goethe University Frankfurt, University Hospital, Department of Nuclear Medicine, Clinic for Radiology and Nuclear Medicine, Frankfurt, Germany.; 2 Nuclear Medicine, Faculty of Medicine, University of Augsburg, Augsburg, Bavaria 86156, Germany.; 3 Molecular Imaging and Therapeutics, Department of Radiology, University of North Carolina, Chapel Hill, NC, USA.; 4 Department of Internal Medicine 4 - Nephrology, Goethe University Frankfurt, Frankfurt am Main, Germany.; 5 Else Kroener-Fresenius-Zentrum for Nephrological Research, Goethe University Frankfurt, Frankfurt am Main, Germany.; 6 Department of Nuclear Medicine and Comprehensive Heart Failure Center, University Hospital Würzburg, Würzburg, Germany.; 7 Faculty of Medicine, Dentistry and Pharmaceutical Sciences, Okayama University, Okayama, Japan.; 8 Division of Nuclear Medicine and Molecular Imaging, The Russell H Morgan Department of Radiology and Radiological Sciences, Johns Hopkins University School of Medicine, Baltimore, MD, USA.; 9 DZHK (German Centre for Cardiovascular Research), Partner Site Frankfurt Rhine-Main, Frankfurt, Germany.

**Keywords:** cardiorenal, renocardiac, heart-kidney axis, molecular imaging, theranostics, PET, cardiorenal syndrome, organ-organ interaction

## Abstract

Cardiorenal syndrome (CRS) involves bidirectional crosstalk between the failing heart and the kidneys. Depending on the *primum movens* (primary cardiac or renal injury), systems-based interactions in the secondary affected organ may include pro-fibrotic signaling, overzealous inflammation, impaired nerve integrity or overactivity of specific renal transporters mediating glucose absorption. Those pathophysiological pillars can be investigated by molecular imaging using SPECT or PET agents. Targeted whole-body molecular imaging may allow for a) systems-based analysis along the heart-kidney axis, b) may provide prognostic information on longitudinal organ-based functional decline or c) may be used for guidance of reparative intervention based on peak activation identified on PET (paradigm of cardiorenal theranostics). We will discuss the current state of translational molecular imaging for CRS, along with future clinical aspects in the field.

## Introduction

Cardiorenal syndrome (CRS) involves bidirectional crosstalk between the failing heart and the kidneys, with renocardiac syndrome (RCS) describing the opposite pathophysiological pathway 1. In recent years, multiple pathophysiological pillars have been identified that initiate and perpetuate deteriorating cardiac or renal function and those include, but are not limited to neurohumoral dysregulation, overzealous inflammation, glucose absorption and fibrotic remodeling 1.

Determining severity and extent of alterations on a subcellular level in both the heart and the kidneys poses a challenge for the referring cardiologist or nephrologist 1. As a gold standard, invasively derived specimens obtained by organ biopsies may be difficult to obtain, are prone to sampling errors, can harm the patient and most often, cannot be repeated - thereby not allowing for longitudinal assessment 2. Blood-based (liquid) biomarkers can address those issues, as they can be collected relatively non-invasively and even be applied for serial follow-up, but provide no insights on a tissue level 2. In this regard, varying conventional imaging modalities have already been investigated, which are characterized by respective strengths and limitations. For instance, ultrasound cannot separate between inflammation and fibrotic changes in the kidneys 3, while computed tomography only provides morphological information. On magnetic resonance imaging (MRI), however, renal inflammation is characterized by increasing apparent diffusion coefficient values 4 and this imaging technique can also determine fibrotic changes in the heart and kidneys 5. Nonetheless, other afore-mentioned pathophysiologic pillars in CRS cannot be assessed by MRI, such as neurohumoral alterations or glucose-mediated metabolic changes. However, based on the injected radionuclide, molecular imaging has the potential to interrogate all of those molecular characteristics in a non-invasive manner. Moreover, as radiotracers are applied systemically, this imaging technique can determine organ-organ crosstalk, including heart and kidneys 6.

In recent years, various therapeutic strategies targeting inflammation, neurohumoral or metabolic alterations in CRS have been developed and yielded mixed results. For instance, in the CARRESS-HF trial (Cardiorenal Rescue Study in Acute Decompensated Heart Failure), ultrafiltration applied to CRS patients with heart failure (HF) and worsening kidney function failed to preserve the renal status quo when compared to intravenous diuretics adjusted to daily urine output 7. Other major studies, however, have focused on renal inhibition of sodium-glucose cotransporter 2 inhibitor (SGLT2) in the context of CRS, including DECLARE-TIMI 58 (Dapagliflozin Effect on CardiovasculAR Events), CANVAS (Canagliflozin Cardiovascular Assessment Study Program), and EMPA-REG OUTCOME (Empagliflozin, Cardiovascular Outcomes, and Mortality in Type 2 Diabetes) 8-10. For the latter trial, a relevant risk reduction of 44% for the doubling of serum creatinine levels was observed 10, thereby delaying the need for dialysis by approximately 1 year 11. Furthermore, SGLT2 inhibition by dapagliflozin or empagliflozin significantly reduced the risk for chronic kidney disease (CKD) progression as well as HF events in patients with CKD with or without diabetes 12. However, despite such optimal treatment including inhibition of renin-angiotensin system or SGLT2, residual risk remains high. Among others, the human monoclonal antibody directed towards IL-1ß also reduced major cardiovascular events (MACE) in patients with CKD, thereby indicating a cardioprotective effect in RCS, but failed to reduce the number of renal events 13. Taken together, the varying therapeutic response in CRS patients highlight an unmet need to non-invasively identify the compromised target along the cardiorenal axis and to initiate the most appropriate treatment for a dual-organ benefit at the right time. In this regard, molecular imaging allows quantification of the current retention capacities of the target in-vivo 6, which then also opens opportunities for molecular image-piloted strategies. Reparative interventions can be commenced at the time of maximum target expression (derived by PET radiotracer uptake), which then allows maximization of therapeutic efficacy and minimization of relevant off-target effects 6. As such, the increasing availability of potential integrated applications of therapy and molecular imaging may then be exploited for a) systems-based analysis along the heart-kidney axis, b) prognostic information on longitudinal organ-based functional decline or c) guidance of reparative intervention based on peak activation identified on PET (paradigm of *cardiorenal theranostics*).

**Figure 1:**
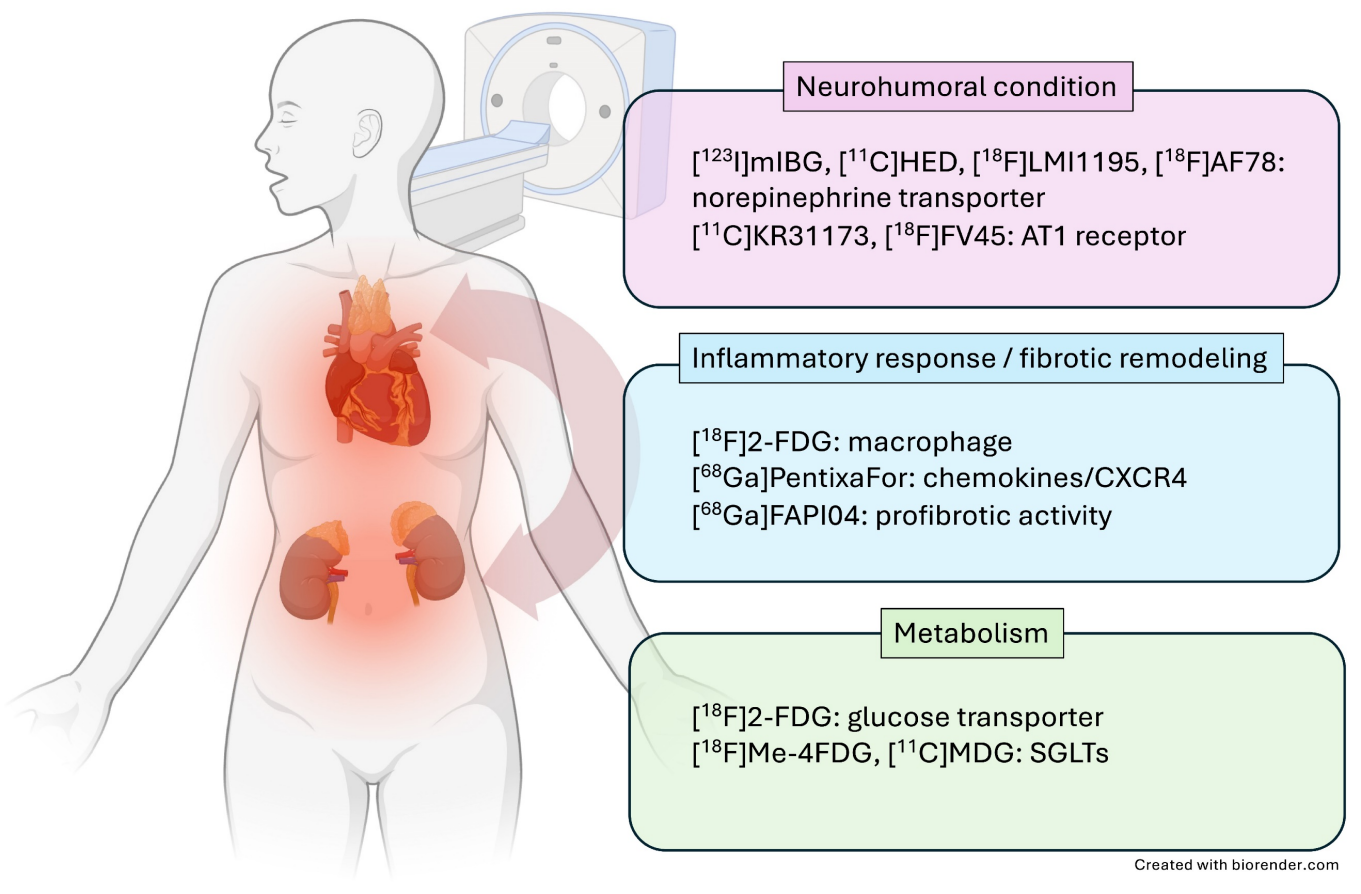
**Overview of selected radiotracers used to decipher target expression along the heart-kidney axis.** [^123^I]-*meta*-iodobenzylguanidine ([^123^I]mIBG), [^11^C]meta- hydroxyephedrine ([^11^C]HED), F18 N-[3-Bromo-4-(3-[^18^F]fluoro-propoxy)-benzyl]-guanidin ([^18^F]LMI1195), 2-Butyl-5-[^11^C]methoxymethyl-6-(1-oxopyridin-2-yl)-3-[[2-(1H-tetrazol-5-yl)biphenyl-4-yl]methyl]-3H-imidazo[4,5-b]pyridine ([^11^C]KR31173), ^18^F-labeled valsartan ([^18^F]FV45), *2*-*deoxy*-2-[^18^F]fluoro-*D*-*Glucose* ([^18^F]FDG), ^68^Ga-labeled FAP inhibitor ([^68^Ga]FAPI04), lpha-methyl-4-deoxy-4-[^18^F]fluoro-D-glucopyranoside ([^18^F]Me-4FDG), 11C-methyl-D-glucoside ([^11^C]MDG). Created with biorender.com.

In the following review article, we will discuss pathophysiological pillars involved in CRS and provide a brief overview of SPECT and PET radiotracers that can visualize respective targets on a subcellular level along the heart-kidney axis (**Figure 1**). Moreover, we will discuss future applications of this imaging technique for CRS patients in clinical practice.

## Inflammation and Pro-Fibrotic Remodeling

In patients after myocardial infarction (MI) and HF exerting renodepressing effects, normal tissue repair is characterized by initiation of inflammatory pathways, followed by pro-fibrotic cells triggering wound healing processes in the heart and kidneys. Prolonged activation of pro-inflammatory/-fibrotic cells can exacerbate acute kidney injury (AKI) post-MI (2). Along the cardiorenal axis (CRA), the healing characteristics of recruited phagocytes may then be self-perpetuating, culminating in deleterious effects. Relative to controls, plasma of CRS Type 1 patients (defined as acute HF leading to AKI) revealed a distinctive time line of early increase of pro-inflammatory cytokines including interleukin (IL)-6 and tumor necrosis factor (TNF)-α 14. Moreover, compatible with CRS Type 1, MI in mice triggered an acute rise in creatinine, which was accompanied by an increased renal expression of pro-inflammatory molecules including vascular cell adhesion molecule-1 (VCAM-1), and transforming growth factor (TGF)-β. That, however, was in contrast to late-stage chronic HF resulting in CKD (CRS Type 2). Mice euthanized later in the time-course showed increasing periglomerular and peritubular fibrosis in the kidneys with increased markers of collagen deposition, most likely caused by oxidant stress resulting in fibroblast activation 15. Accordingly, anti-inflammatory therapies emerged as a novel therapeutic option in patients with cardiorenal diseases 16. In patients, modulation of inflammation has been recently tested in a post-hoc analysis of the CANTOS (Canakinumab Anti-Inflammatory Thrombosis Outcomes Study) trial, which was designed as an intent-to-treat analysis in 10,061 adult patients with recent MI and systemic inflammation (defined as elevated high sensitivity c-reactive protein [hsCRP] >2 mg/L). Patients with moderate CKD (estimated glomerular filtration rate [eGFR] <60 ml/min/1.73 m2) treated with the IL-1β selective antagonist canakinumab demonstrated a significantly reduced risk of MACE. The largest benefit was observed in patients with a robust anti-inflammatory response to the first dose (on-treatment hsCRP levels <2 mg/l), thereby emphasizing the critical role of appropriate patient selection and timing of treatment 13. Besides that, Ziltivekimab a human monoclonal antibody directed against the IL-6 ligand is currently evaluated in the phase 3 clinical trial ZEUS in patients with CKD, prevalent atherosclerotic cardiovascular disease, and elevated CRP 17. Hence, a more sophisticated clinical strategy of limiting anti-inflammatory therapy exclusively to responders might improve therapeutic efficacy for bifunctional cardiorenal outcome 18.

**Figure 2:**
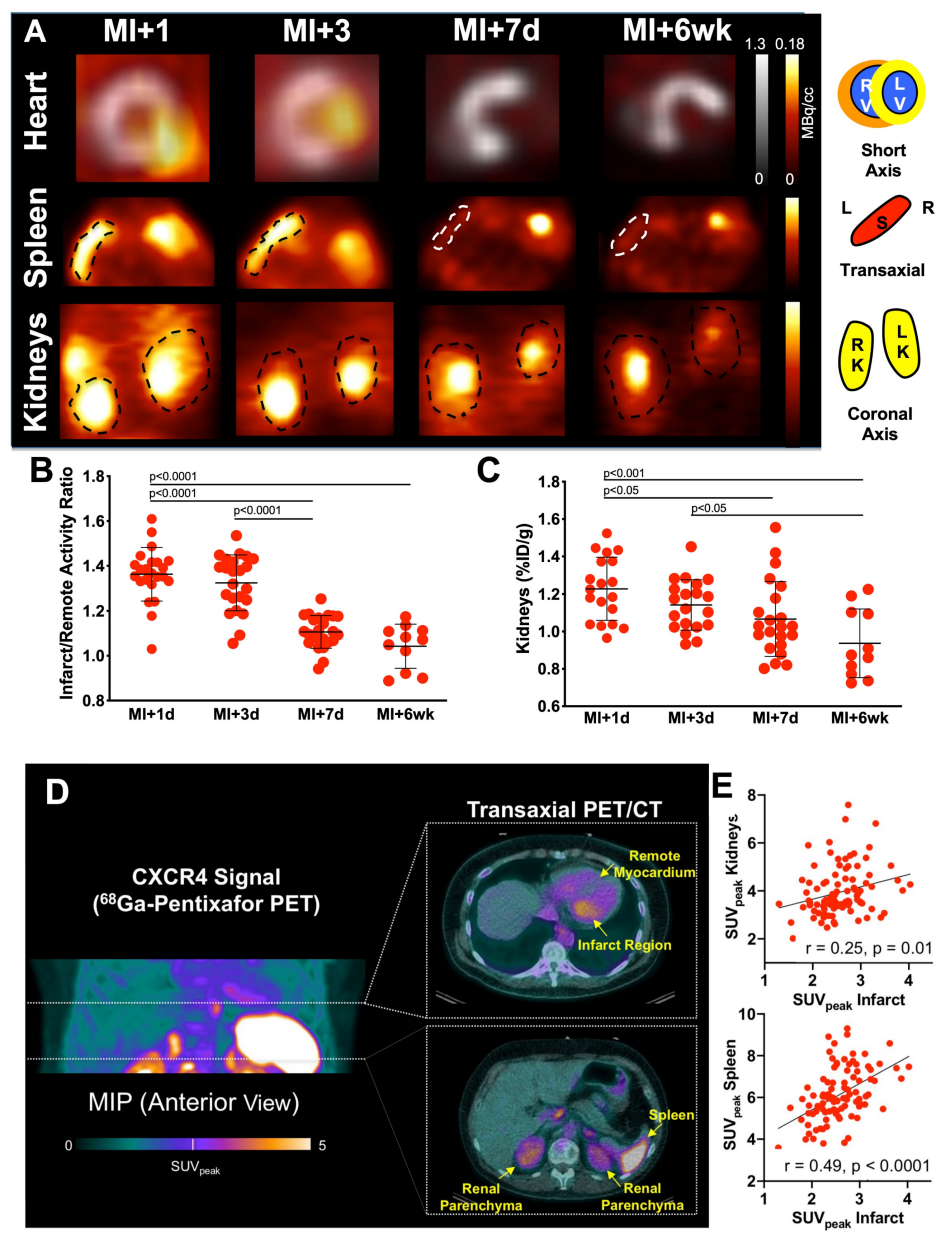
** Cardiorenal C-X-C motif chemokine receptor 4 (CXCR4)-directed PET imaging using [^68^Ga]PentixaFor in mice (A-C) and patients (D, E) after myocardial infarction (MI). (A)** [^68^Ga]PentixaFor revealed intense CXCR4 uptake early after MI (day [d] 1, day 3) in the infarct territory (short axis) and no relevant signal at later stages (day 7, week [wk] 6). Respective splenic and renal images (coronal axis) showed paralleled radiotracer decline over time. Quantitative evaluation provided by myocardial infarct to remote activity ratio **(B)** and renal [^68^Ga]PentixaFor signal (in %ID/g, **C**) confirmed visual findings with significant decline of in-vivo CXCR4 expression already at d7 relative to d1 post-MI. Maximum intensity projection (MIP) of [^68^Ga]PentixaFor in a patient early after MI revealed intense uptake in the infarct region, spleen and parenchyma of the kidneys **(D)**. Peak standardized uptake values (SUV_peak_) in the infarct region showed significant association with renal and splenic uptake** (E)**, indicative for systemic immune response (as demonstrated by hematopoietic organ activation) and cardiorenal inflammatory interaction. Right ventricle (RV), left ventricle (LV), Spleen (S), L (Left), R (Right), K (Kidney). Adapted with permission from 24, copyright 2021 IvySpring.

In recent years, the C-X-C motif chemokine receptor 4 (CXCR4) and its known natural ligand SDF1-alpha have gained attention, as they mediate migration of leukocyte subpopulation to the injured myocardium post-MI 19. Thackeray and coworkers reported on the use of CXCR4-targeting PET probe [^68^Ga]PentixaFor in a murine MI model, demonstrating increased uptake in area of ischemic injury 3 days after the acute event, followed by dissipation of the biomarker signal at day 7, which was also correlated to leukocyte infiltration determined by flow cytometry 20. The same research group observed improved left ventricular ejection fraction (LVEF) in mice treated at the peak PET signal at day 3, but not at day 7, indicative of the capability of chemokine receptor PET to guide anti-inflammatory cardiac repair 21. In human, the CXCR4 PET signal varied substantially among patients, also suggesting that CXCR4-targeted molecular imaging can identify patients that benefit from treatment 21. Those considerations are further fueled by a recent study showing that patients with increased i*n vivo* CXCR4 expression in the area of ischemic injury were also at greater risk for developing MACE during follow-up 22. Chemokine receptor PET, however, allows for systemic read-out of other organs involved in the systemic immune response, and thus, in mice after MI, radiotracer uptake in the bone marrow, spleen or lymph nodes was proportional to [^68^Ga]PentixaFor uptake in the infarct territory 22. The kidneys, however, are also characterized by increased leukocyte infiltration 23 and thus, a recent translational study investigating parallel heart-kidney inflammation after acute MI using CXCR4-directed PET was carried out. Murine CXCR4 PET signal in the injured myocardium correlated with renal radiotracer accumulation (**Figure 2A-C**), which was proportional to CD68 positive macrophages 24. Potentially providing evidence of the subsequent on-set of inflammation in both organs over time, the renal CXCR4 PET uptake at 7 days post-MI emerged as more predictive of functional cardiac decline (assessed by LVEF) when compared to the infarct signal 24. In 96 patients with MI who were imaged with CXCR4-directed PET immediately after the acute event, comparable cardiorenal systemic networking was observed, as CXCR4 signal in the infarct territory was linked to renal and splenic radiotracer accumulation (**Figure 2 D,E**). Moreover, preliminary data also showed that the cardiac PET signal may identify patients at increased risk for worsening renal function post-MI, suggesting a potential role for image-piloted therapeutic approaches to preserve (cardio)renal function after acute MI 24. Further evidence on a systems-based organ-organ networking analysis based on inflammatory-directed imaging will be provided by results of a currently recruiting prospective phase 2 study, which will image optimally treated acute MI patients using [^68^Ga]PentixaFor PET to determine the predictive value of organ uptake (including kidneys) for LVEF decline during one-year follow-up (LOMI trial, NCT05519735).

In deciphering (pro-)fibrotic expression, recent years have seen an unprecedented success of fibroblast activation protein (FAP)-directed PET agents, many of which are FAP inhibitors (FAPI), mainly in oncology 25. After acute MI, overzealous inflammatory response can cause aggregation of structural proteins of the extracellular matrix, followed by fibrotic remodeling 26. After approximately 11 days post-MI, a recent human study reported on increased FAPI uptake in the ischemic myocardium. The fibrotic PET signal exceeded late gadolinium enhancement signal and was negatively associated with later LVEF, indicating that FAPI-targeted imaging can provide additional information beyond conventional morphological modalities and may be of predictive value 27. A recent study investigated the potential of FAPI PET to identify fibrosis, thereby potentially omitting invasive procedures for the detection of kidney fibrosis. In a head-to-head comparison with other non-fibrotic PET radiotracers, only [^68^Ga]FAPI uptake in the renal parenchyma was inversely proportional to GFR values, suggesting a specific binding for this PET agent in the renal cortex 28. Also demonstrating the potential of monitoring kidney fibrosis, Unterrainer *et al.* reported on a patient after renal artery occlusion who exhibited increased fibrotic uptake in an (ischemic) area at risk at the lower-pole of the kidney. *Ex vivo* analysis then revealed activated fibroblasts in this region 29. Although investigations along the cardiorenal axis are missing to date, those studies in the infarcted heart and kidney suggest that FAPI PET may serve as a biomarker of the fibrotic extent and remodeling in both organs after myocardial or renal infarction. The role of fibrosis-targeting radiotracers may then include evaluation of fibrotic changes in the kidneys to guide novel renal fibrosis decreasing therapeutics, e.g., the transforming growth factor-inhibiting GW788388 30. As such, FAPI PET could then close the gap between potential preclinical anti-fibrotic phenotypes and beneficial treatment in patients, as preceeding interrogation of renal fibrosis in both organs would allow for improved timing of treatment 31.

## Neurohumoral Dysregulation

After acute MI, neurohumoral alterations can substantially contribute to HF, including overactivity of the sympathetic nervous system (SNS) and renin angiotensin-aldosterone system (RAAS) 32. After MI, the neurotransmitter norepinephrine (NE) is not consistently cleared from the synaptic cleft at the nerve terminal, which then contributes to remodeling of the left ventricle, followed by the development of congestive HF (CHF) 32. In addition, dysregulation of the SNS and RAAS after cardiac damage is also thought to play a prominent role in exacerbating renal impairment, including hypo-uresis 1.

**Figure 3:**
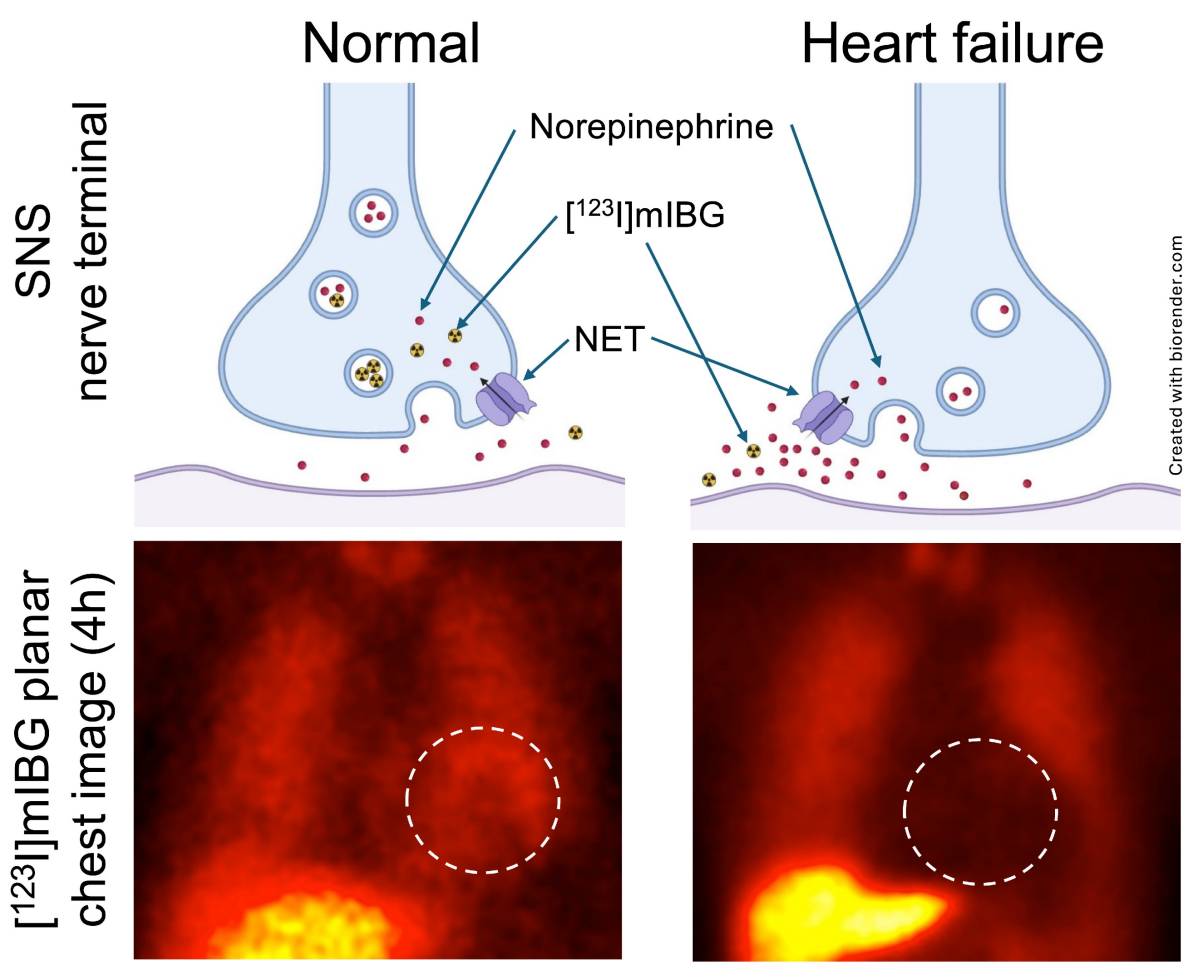
** Norepinephrine (NE) transporter (NET)-directed molecular using [^123^I]mIBG planar scintigraphy.** In healthy patients (upper row, left), NE is cleared from the synaptic cleft in presynaptic nerve terminal once a firing impulse has arrived. In heart failure patients, neurotransmission is characterized by a compromised reuptake mechanism (upper row, right). Mimicking the physiological pathway of NE, the [^123^I]mIBG signal is then diminished in HF patients (lower row, right), while the radiotracer signal is intense in healthy individuals (lower row, left). Dotted circles indicate myocardium. SNS = sympathetic nervous system. Created with biorender.com.

Cardiac innervation can be closely monitored by means of molecular imaging. After storage in presynaptic NE vesicles, the firing impulse at the nerve terminal causes extraction into the synaptic cleft, leading to further neurotransmission via postsynaptic adrenoreceptors. This process can be visualized by radiotracers imitating this physiological route of NE, such as the SPECT radiotracer [^123^I]-*meta*-iodobenzylguanidine ([^123^I]mIBG) 33. In patients with HF, the disrupted neurotransmission and missing reuptake via the NE transporter (NET) into the pre-synapse is then characterized by absent radiotracer accumulation in the myocardium (**Figure 3**). Globally assessed impaired cardiac nerve integrity using SPECT technology then allows for identifying patients at increased risk for fatal events in HF 34. Aiming to identify potential cardiorenal interaction in the failing heart, Verschure *et al.* showed that [^123^I]mIBG-determined cardiac nerve integrity emerged as a better predictor for cardiac death than GFR 35. Marsico and coworkers recently reported that increased myocardial denervation assessed by [^123^I]mIBG was associated with more severe GFR reduction when compared to subjects with normal renal function, thereby further emphasizing the potential of SPECT to decipher neurohumoral cardiorenal crosstalk 36. Amami *et al.* also identified a predictive role of [^123^I]mIBG scintigraphy and renal function. In CHF patients, abnormal cardiac nerve function along with diagnosis of CKD was most predictive for potentially fatal arrhythmic events 37.

Relative to SPECT, however, ^18^F-labeled PET radiotracers allow for more precise assessment of the reduced cardiac innervation, mainly due to improved spatiotemporal resolution 38. Of note, recent years have witnessed the introduction of those second-generation molecular imaging radiotracers for assessing neurohumoral function, including a non-invasive read-out of the current status of the RAAS and SNS. For the latter system, the novel PET probe [^18^F]AF78 provided a precise reflection of NET activity, as the NET blocking agent phenoxybenzamine led to diminished uptake in the myocardium 39. Based on the clinically used drug valsartan, the radiotracer ω-fluoro-valsartan ([^18^F]FV45) targeting the RAAS allows for determination of AT1 receptor expression. In this regard, valsartan was used as a “cold” blocking agent in rats, which led to diminished renal [^18^F]FV45 accumulation, thereby suggesting selective interaction of this radiotracer with the AT1 receptor in the kidneys 40. Although human reports are missing to date, those promising results render [^18^F]FV45 as a promising second-generation ^18^F-labeled PET radiotracer for deciphering neurohumoral interaction along the heart-kidney axis.

## SGLT2 - Beyond Glucose Lowering

Sodium-glucose cotransporter-2 (SGLT2) inhibitors have demonstrated benefits that extend beyond glucose-lowering efficacy, including protective mechanisms capable of slowing or preventing the onset of long-term cardiovascular and renal complications 41. SGLT2 is a protein located in the proximal tubules of the kidneys, responsible for the reabsorption of the majority of filtered glucose from the renal tubular lumen back into the bloodstream 42. Respective inhibitors can cause increased excretion of glucose in the urine and subsequently lowering blood glucose levels.

SGLT2 inhibitors, such as empagliflozin and dapagliflozin, have shown significant cardiovascular benefits in multiple large-scale cardiovascular outcome trials 43. These benefits encompass reductions in MACE, hospitalization for HF, and cardiovascular mortality. SGLT2 inhibitors induce natriuresis and osmotic diuresis, reducing preload and afterload, thereby improving cardiac function 44. Additionally, by reducing glucotoxicity, SGLT2 inhibitors improve insulin sensitivity and β-cell function, mitigating the adverse effects of hyperglycemia on the cardiovascular system 45. These inhibitors also reduce inflammation and fibrosis in cardiac tissues, contributing to improved cardiovascular outcomes 46.

In terms of renal protection, SGLT2 inhibitors provide significant benefits as evidenced by reductions in the progression of CKD and the occurrence of renal-related adverse events in CKD patients with or without diabetes 47. By enhancing sodium delivery to the macula densa, SGLT2 inhibitors restore tubule-glomerular feedback, reducing intraglomerular pressure and slowing CKD progression 48. They also reduce albuminuria, a marker of glomerular damage, thereby preserving kidney function 49. Furthermore, the diuretic effect of SGLT2 inhibitors alleviates renal hypoxia, protecting against ischemic damage 50.

**Figure 4:**
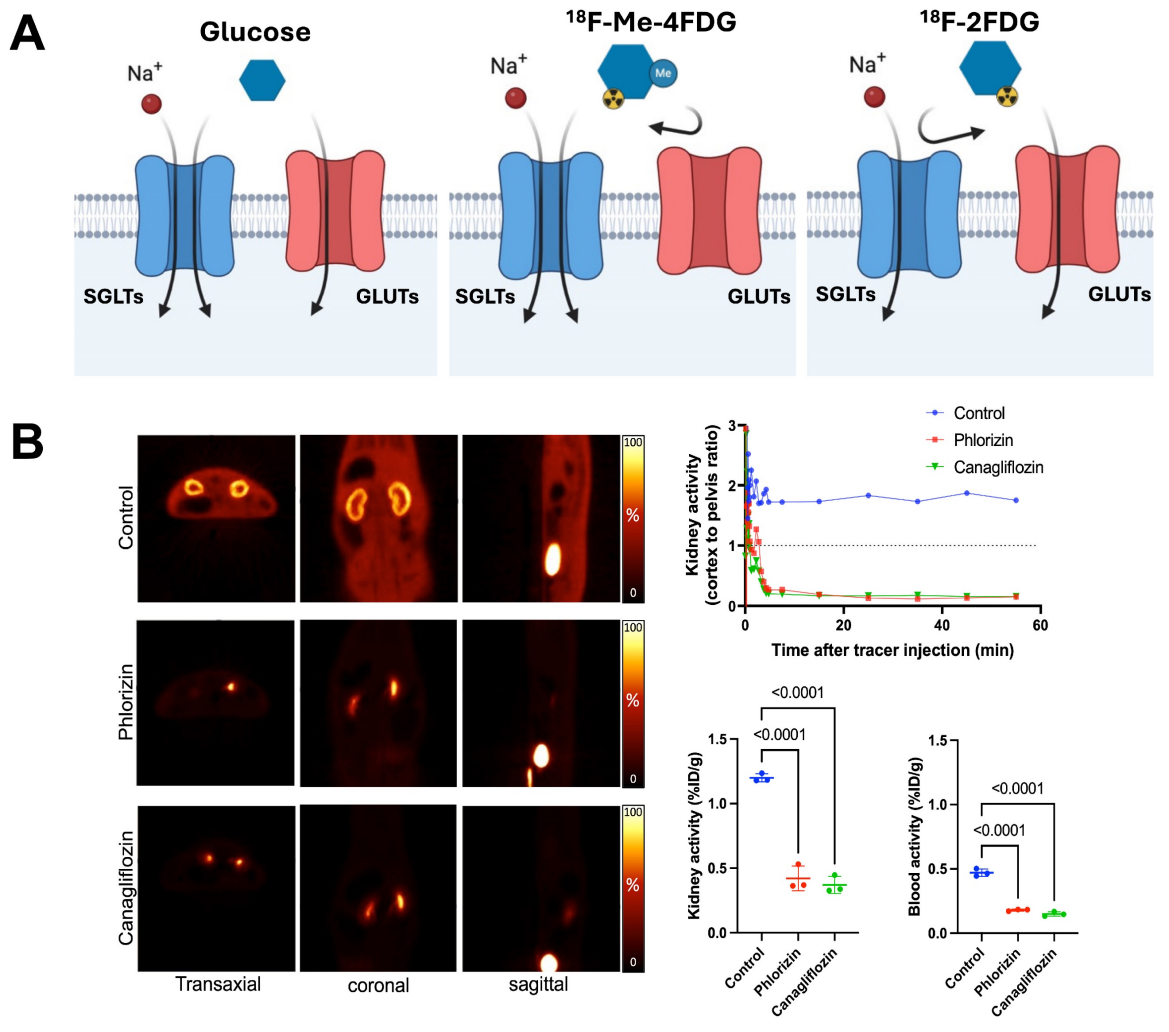
** SGLT-directed molecular imaging using [^18^F]Me-4FDG. (A)** Relative to [^18^F]FDG (right panel), which interacts with GLUT, [^18^F]Me-4FDG is selectively taken up by SGLT (middle panel). **(B)** Renal [^18^F]Me-4FDG 10-20 min after i.v. injection (in healthy rats (left panels). Stable radiotracer accumulation was observed in controls, while in Phlorizin- and Canagliflozin-pretreated animals, uptake in the cortex was substantially decreased (along with high bladder activity). Cortex-to-pelvis count ratios (upper row, right) also displayed lower radiotracer accumulation over time relative to controls. Kidney and blood radioactivity (lower row, right) at 60 min after tracer i.v. showed increased renal activity only for controls, but not for pretreated animals, thereby indicating high specificity of [^18^F]Me-4FDG for deciphering SGLT activity in-vivo. Adapted and changed with permission from 51, copyright 2022 Sage. Published under Creative Commons Attribution 4.0 License (https://creativecommons.org/licenses/by/4.0/).

The integration of advanced molecular imaging techniques, including cutting-edge molecular PET imaging with SGLT-specific radiotracers and other PET agents, offers a comprehensive approach to assessing the multifaceted benefits of SGLT2 inhibitors. These imaging modalities allow for the precise evaluation of neurohumoral, metabolic, and inflammatory processes (**Figure 1**), providing deeper insights into the mechanisms of action and therapeutic effects of SGLTs inhibitors. The development of the PET agent alpha-methyl-4-deoxy-4-18F-fluoro-D-glucopyranoside ([^18^F]Me-4FDG) has enabled non-invasive measurement of SGLT activity *in-vivo* (**Figure 4**). [^18^F]Me-4FDG, unlike the widely used *2*-*deoxy*-2-[^18^F]fluoro-*D*-*Glucose* ([^18^F]FDG), is selectively taken up by SGLTs but not by glucose transporters (GLUTs), providing a targeted approach to visualize and quantify SGLTs activity 51. Once inside the cell, [^18^F]Me-4FDG is phosphorylated by hexokinase and remains trapped, allowing for imaging of its distribution and concentration. This imaging can visualize and quantify renal SGLT2 activity, potentially allowing for precise monitoring of SGLT2 inhibitor efficacy in reducing glomerular hyperfiltration and protecting against CKD progression 51.

Molecular imaging agents targeting the cardiac neurohormonal system, including the aforementioned SPECT radiotracer [^123^I]mIBG and the recently introduced fluorine-18 NE analog PET radiotracer [^18^F]AF78 can be used to assess the impact of SGLT2 inhibitors on cardiac neurohumoral regulation 52. Those agents visualize SNS activity, providing insights into the effects of SGLT2 inhibitors on cardiac autonomic regulation. Metabolic imaging with tracers like [^18^F]FDG for glucose metabolism (through binding of GLUTs) and fatty acid markers such as ^18^F-fluoro-4-thia-palmitate ([18F]FTP), 18-[^18^F]fluoro-4-thia-oleate ([^18^F]FTO) PET or [^123^I]beta-methyl iodophenyl-pentadecanoic acid ([^123^I]BMIPP) SPECT can complement SGLT2-specific imaging by providing a broader view of metabolic changes induced by SGLT2 inhibitors 53, 54. Those tracers can evaluate myocardial metabolism, offering comprehensive insights into the metabolic effects on cardiac function. As alluded to earlier, for inflammation imaging, tracers such as the CXCR4-targeting PET tracer [^68^Ga]PentixaFor can visualize and quantify inflammation in the injured heart and kidneys post-MI 24. That can help assess the anti-inflammatory effects of SGLT2 inhibitors, further elucidating their role in reducing cardiovascular and renal complications. SGLT2 inhibitors represent a transformative advancement in the management of diabetes, with profound benefits for cardiovascular and renal health; as such, mechanistic insights from molecular imaging of the appropriate pathways and targets may be impactful in optimizing diabetes care.

**Table 1** provides an overview of selected radiotracers and target mechanisms used along the cardiorenal axis.

## The Paradigm of Cardiorenal Theranostics

The American Heart Association emphasized the need for novel imaging techniques for improved selection and goal-directed reparative interventions for patients with CRS 1. Given varying pathophysiological pillars along the heart-kidney axis that can be characterized by means of molecular imaging, SPECT or PET-piloted therapeutic strategies may improve outcome in MI or HF patients not only for the primarily affected organ (heart), but also for the remote kidneys.

**Figure 5:**
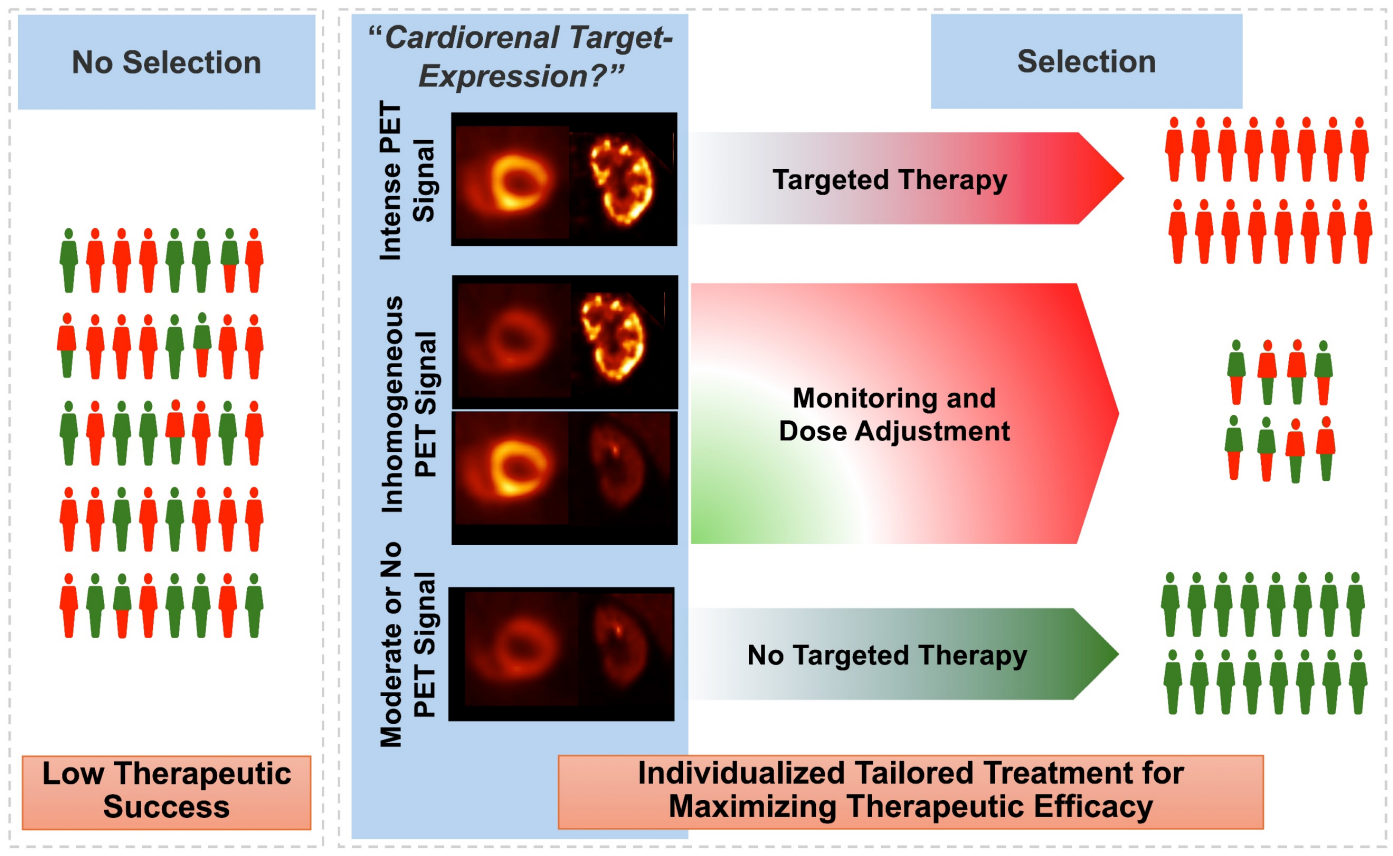
** Paradigm of cardiorenal theranostics.** In a manner similar to oncology, the concept of theranostics could be transferred to individuals with cardiorenal syndrome. Patients with increased radiotracer signal, e.g., after injection of inflammatory-directed [^68^Ga]PentixaFor, will receive targeted, anti-inflammatory therapy, thereby improving cardiorenal functional outcome. For patients with diminished PET signal in both target organs, other treatment options should be identified. As such, PET can then allow for a molecular image-piloted strategies to determine the most appropriate patient at the right time for the right treatment. Inconsistencies on cardiorenal uptake may be addressed by dose-depending therapeutic approaches, e.g., lower uptake may trigger lower dose of a cardiorenal protective agent.

In nuclear oncology, recent years have seen an unprecedented success of theranostics, where the target on tumor cell surface can be visualized by PET, followed by treatment using a comparable therapeutic radiotracer 55. This principle could also be transferred to CRS, with target-specific PET agents deciphering the current status of the receptor that would also be occupied for therapeutic purposes 56. For instance, patients with increased signal on inflammatory PET on both the heart and the kidneys could then be scheduled for anti-inflammatory drugs, such as canakinumab. As such, relative to a blood-based identification of responders (e.g., by measuring CRP) 13, such a molecular imaging-based phenotyping would then allow the identification of subjects with best response. That paradigm of “cardiorenal theranostics” would then allow for highly specific targeted intervention, thereby maximizing functional outcome of both organs (**Figure 5**). Nonetheless, molecular imaging can always be only one selection tool among others, as therapeutic outcomes may be influenced by various factors, including individual patient differences, the resolution limitations of imaging techniques, and the timing of treatment.

## Current Limitations and Outlook

Obviously, some of the herein mentioned radiotracers have been limited to one single organ to date. The reasons for this development are manifold: First, the afore-mentioned radiotracers targeting inflammation and neurohumoral integrity have a long(er)-standing history relative to other radiopharmaceuticals. For instance, the first report on [^123^I]mIBG dates back to 1980 57, while the first in-human data on FAPI was presented in 2018 58. Second, some of those radiotracers are limited to particular PET centers 39, while other “target of interests” such as SGLT have just recently gained attention 59. To ensure that PET and SPECT become truly useful for molecular imaging along the heart-kidney axis, a close collaboration between cardiologists, nephrologists and molecular imaging experts focusing on CRA would be essential. Such a multidisciplinary approach would then ensure that the next important target for treatment along the heart-kidney axis would be accompanied by a respective imaging agent, which allows for an organ-specific phenotyping. Moreover, the use of SPECT and PET has been exclusively focused on cardiorenal interactions with the injured myocardium serving as “primum movens” and thus, future efforts should also include molecular imaging along the renocardiac axis (i.e., focusing on diseases with renal damage followed by heart injury). Last, the current use of this imaging technique relative to other imaging methods needs to be further defined and thus, the role of PET/SPECT along the CRA may be further characterized by head-to-head comparisons, preferably in clinical trials.

## Conclusions

In patients with CRS, systemically applied SPECT and PET radiotracers can provide information on a subcellular level for the target (heart) and remote organ (kidneys) and may even be used to guide reparative interventions along the CRA (i.e. a paradigm of cardiorenal theranostics). Respective imaging agents can decipher pro-fibrotic signaling, overzealous inflammation, impaired nerve integrity, and/or overactivity of specific renal transporters mediating glucose absorption. For instance, translational studies have already reported parallel heart-kidney inflammation after acute MI using chemokine receptor-directed PET, while SPECT-based neurohumoral impairment was tightly linked to greater risk of renal functional decline or lethal arrhythmic events. Future efforts may turn towards the use of novel ^18^F-labeled second-generation SNS or RAAS radiotracers for identifying CRS patients at greater risk for cardiorenal functional decline. Moreover, SGLT2 activity reflecting PET radiotracers may also allow for precise monitoring of SGLT2 inhibitor efficacy in reducing glomerular hyperfiltration.

## Funding

German Research Foundation (453989101, TH, RAW; 507803309, RAW); Okayama University (RECTOR Program, TH); Japan Society for the Promotion of Science (23K24288, TH).

## Figures and Tables

**Table 1:** ** Overview of selected radiotracers and target mechanisms used along the cardiorenal axis.** Cardiorenal axis (CRA), ^68^Ga-labeled FAP inhibitor ([^68^Ga]FAPI04), sympathetic nervous system (SNS), [^123^I]-*meta*-iodobenzylguanidine ([^123^I]mIBG), renin angiotensin-aldosterone system (RAAS), F18 valsartan ([^18^F]FV45), lpha-methyl-4-deoxy-4-[^18^F]fluoro-D-glucopyranoside ([^18^F]Me-4FDG).

Target		Radionuclide	Current use
Inflammation	**Chemokine Receptor 4**	[^68^Ga]PentixaFor	**Preclinical monitoring along the CRA:** Murine MI model with increased CXCR4 expression early post-infarct 20, improved cardiac outcome with treatment at peak of PET signal 21 and paralleled heart-kidney CXCR4 expression along the cardiorenal axis 24 **Clinical monitoring along the CRA**: Paralleled heart-kidney inflammation in MI patients early after the acute event, with independent predictive value of PET signal for worsening kidney function 24
Fibrosis	**Fibroblast activation protein inhibitor**	[^68^Ga]FAPI	**Predictive value after MI**: Increased FAPI uptake in the ischemic myocardium 11 days after MI on patients; uptake associated with later changes of ejection fraction, thereby suggesting predictive potential 27 **Monitoring of renal function**: FAPI uptake in the renal parenchyma inversely proportional to GFR values, suggesting a specific binding for this PET agent in the kidneys 28 **Monitoring of renal fibrosis**: after renal artery occlusion 29
Neurohumoral Dysregulation	**Norepinephrine Transporter (NET) for SNS assessment**	[^123^I]mIBG	Increased uptake **associated** with worsening kidney function 36 **Predictive role of combined cardiorenal assessment:** [123I]-mIBG cardiac innervation scintigraphy and renal function can predict lethal arrhythmic events 37
		[^18^F]AF78	Preclinical: **selective interaction with the NET** in the heart as proven by blocking experiments in rats 39
	**AT1 Receptor for RAAS assessment**	[^18^F]FV45	Preclinical: **selective interaction with the AT1 receptor** in the kidneys as proven by blocking experiments in rats 40
Metabolism	**Sodium-glucose cotransporter-2 (SGLT2)**	[^18^F]Me-4FDG	Preclinical: **selectively taken up by SGLT** but not by GLUT as proven by blocking experiments in rats 51

## References

[1] Rangaswami J, Bhalla V, Blair JEA, Chang TI, Costa S, Lentine KL (2019). Cardiorenal Syndrome: Classification, Pathophysiology, Diagnosis, and Treatment Strategies: A Scientific Statement From the American Heart Association. Circulation.

[2] Heitzer E, Perakis S, Geigl JB, Speicher MR (2017). The potential of liquid biopsies for the early detection of cancer. NPJ Precis Oncol.

[3] Grenier N, Merville P, Combe C (2016). Radiologic imaging of the renal parenchyma structure and function. Nat Rev Nephrol.

[4] Buchanan CE, Mahmoud H, Cox EF, McCulloch T, Prestwich BL, Taal MW (2020). Quantitative assessment of renal structural and functional changes in chronic kidney disease using multi-parametric magnetic resonance imaging. Nephrol Dial Transplant.

[5] Edwards NC, Moody WE, Chue CD, Ferro CJ, Townend JN, Steeds RP (2014). Defining the natural history of uremic cardiomyopathy in chronic kidney disease: the role of cardiovascular magnetic resonance. JACC Cardiovasc Imaging.

[6] Werner RA, Thackeray JT, Diekmann J, Weiberg D, Bauersachs J, Bengel FM (2020). The Changing Face of Nuclear Cardiology: Guiding Cardiovascular Care Toward Molecular Medicine. J Nucl Med.

[7] Bart BA, Goldsmith SR, Lee KL, Givertz MM, O'Connor CM, Bull DA (2012). Ultrafiltration in decompensated heart failure with cardiorenal syndrome. N Engl J Med.

[8] Wiviott SD, Raz I, Bonaca MP, Mosenzon O, Kato ET, Cahn A (2019). Dapagliflozin and Cardiovascular Outcomes in Type 2 Diabetes. N Engl J Med.

[9] Neal B, Perkovic V, Mahaffey KW, de Zeeuw D, Fulcher G, Erondu N (2017). Canagliflozin and Cardiovascular and Renal Events in Type 2 Diabetes. N Engl J Med.

[10] Wanner C, Inzucchi SE, Lachin JM, Fitchett D, von Eynatten M, Mattheus M (2016). Empagliflozin and Progression of Kidney Disease in Type 2 Diabetes. N Engl J Med.

[11] Wanner C (2017). EMPA-REG OUTCOME: The Nephrologist's Point of View. Am J Med.

[12] The E-KCG, Herrington WG, Staplin N, Wanner C, Green JB, Hauske SJ (2023). Empagliflozin in Patients with Chronic Kidney Disease. N Engl J Med.

[13] Ridker PM, MacFadyen JG, Glynn RJ, Koenig W, Libby P, Everett BM (2018). Inhibition of Interleukin-1beta by Canakinumab and Cardiovascular Outcomes in Patients With Chronic Kidney Disease. J Am Coll Cardiol.

[14] Virzi GM, Torregrossa R, Cruz DN, Chionh CY, de Cal M, Soni SS (2012). Cardiorenal Syndrome Type 1 May Be Immunologically Mediated: A Pilot Evaluation of Monocyte Apoptosis. Cardiorenal Med.

[15] Lu J, Wang X, Wang W, Muniyappa H, Deshmukh A, Hu C (2012). Abrogation of lectin-like oxidized LDL receptor-1 attenuates acute myocardial ischemia-induced renal dysfunction by modulating systemic and local inflammation. Kidney Int.

[16] Speer T, Dimmeler S, Schunk SJ, Fliser D, Ridker PM (2022). Targeting innate immunity-driven inflammation in CKD and cardiovascular disease. Nat Rev Nephrol.

[17] Ridker PM (2021). From RESCUE to ZEUS: will interleukin-6 inhibition with ziltivekimab prove effective for cardiovascular event reduction?. Cardiovasc Res.

[18] Sehested TSG, Bjerre J, Ku S, Chang A, Jahansouz A, Owens DK (2019). Cost-effectiveness of Canakinumab for Prevention of Recurrent Cardiovascular Events. JAMA Cardiol.

[19] Wang Y, Dembowsky K, Chevalier E, Stuve P, Korf-Klingebiel M, Lochner M (2019). C-X-C Motif Chemokine Receptor 4 Blockade Promotes Tissue Repair After Myocardial Infarction by Enhancing Regulatory T Cell Mobilization and Immune-Regulatory Function. Circulation.

[20] Thackeray JT, Derlin T, Haghikia A, Napp LC, Wang Y, Ross TL (2015). Molecular Imaging of the Chemokine Receptor CXCR4 After Acute Myocardial Infarction. JACC Cardiovasc Imaging.

[21] Hess A, Derlin T, Koenig T, Diekmann J, Wittneben A, Wang Y (2020). Molecular imaging-guided repair after acute myocardial infarction by targeting the chemokine receptor CXCR4. Eur Heart J.

[22] Werner RA, Koenig T, Diekmann J, Haghikia A, Derlin T, Thackeray JT (2022). CXCR4-Targeted Imaging of Post-Infarct Myocardial Tissue Inflammation: Prognostic Value After Reperfused Myocardial Infarction. JACC Cardiovasc Imaging.

[23] Linhart C, Ulrich C, Greinert D, Dambeck S, Wienke A, Girndt M (2018). Systemic inflammation in acute cardiorenal syndrome: an observational pilot study. ESC Heart Fail.

[24] Werner RA, Hess A, Koenig T, Diekmann J, Derlin T, Melk A (2021). Molecular imaging of inflammation crosstalk along the cardio-renal axis following acute myocardial infarction. Theranostics.

[25] Hirmas N, Hamacher R, Sraieb M, Ingenwerth M, Kessler L, Pabst KM (2023). Fibroblast-Activation Protein PET and Histopathology in a Single-Center Database of 324 Patients and 21 Tumor Entities. J Nucl Med.

[26] Lafuse WP, Wozniak DJ, Rajaram MVS (2020). Role of Cardiac Macrophages on Cardiac Inflammation, Fibrosis and Tissue Repair. Cells.

[27] Diekmann J, Koenig T, Thackeray JT, Derlin T, Czerner C, Neuser J (2022). Cardiac Fibroblast Activation in Patients Early After Acute Myocardial Infarction: Integration with MR Tissue Characterization and Subsequent Functional Outcome. J Nucl Med.

[28] Conen P, Pennetta F, Dendl K, Hertel F, Vogg A, Haberkorn U (2022). [(68) Ga]Ga-FAPI uptake correlates with the state of chronic kidney disease. Eur J Nucl Med Mol Imaging.

[29] Unterrainer LM, Sisk AE Jr, Czernin J, Shuch BM, Calais J, Hotta M (2023). [(68)Ga]Ga-FAPI-46 PET for Visualization of Postinfarction Renal Fibrosis. J Nucl Med.

[30] Petersen M, Thorikay M, Deckers M, van Dinther M, Grygielko ET, Gellibert F (2008). Oral administration of GW788388, an inhibitor of TGF-beta type I and II receptor kinases, decreases renal fibrosis. Kidney Int.

[31] Henderson NC, Rieder F, Wynn TA (2020). Fibrosis: from mechanisms to medicines. Nature.

[32] Zelt JGE, deKemp RA, Rotstein BH, Nair GM, Narula J, Ahmadi A (2020). Nuclear Imaging of the Cardiac Sympathetic Nervous System: A Disease-Specific Interpretation in Heart Failure. JACC Cardiovasc Imaging.

[33] Werner RA, Chen X, Hirano M, Rowe SP, Lapa C, Javadi MS (2018). SPECT vs. PET in cardiac innervation imaging: clash of the titans. Clin Transl Imaging.

[34] Narula J, Gerson M, Thomas GS, Cerqueira MD, Jacobson AF (2015). (1)(2)(3)I-MIBG Imaging for Prediction of Mortality and Potentially Fatal Events in Heart Failure: The ADMIRE-HFX Study. J Nucl Med.

[35] Verschure DO, Somsen GA, van Eck-Smit BL, Verberne HJ (2012). Renal Function in Relation to Cardiac (123)I-MIBG Scintigraphy in Patients with Chronic Heart Failure. Int J Mol Imaging.

[36] Marsico F, Paolillo S, Gargiulo P, Parisi V, Nappi C, Assante R (2021). Renal function and cardiac adrenergic impairment in patients affected by heart failure. J Nucl Cardiol.

[37] Amami K, Yamada S, Yoshihisa A, Kaneshiro T, Hijioka N, Nodera M (2022). Predictive impacts of chronic kidney disease and cardiac sympathetic nervous activity on lethal arrhythmic events in chronic heart failure. Ann Noninvasive Electrocardiol.

[38] Werner RA, Chen X, Rowe SP, Lapa C, Javadi MS, Higuchi T (2020). Recent paradigm shifts in molecular cardiac imaging-Establishing precision cardiology through novel (18)F-labeled PET radiotracers. Trends Cardiovasc Med.

[39] Chen X, Fritz A, Werner RA, Nose N, Yagi Y, Kimura H (2020). Initial Evaluation of AF78: a Rationally Designed Fluorine-18-Labelled PET Radiotracer Targeting Norepinephrine Transporter. Mol Imaging Biol.

[40] Chen X, Hirano M, Werner RA, Decker M, Higuchi T (2018). Novel (18)F-Labeled PET Imaging Agent FV45 Targeting the Renin-Angiotensin System. ACS Omega.

[41] Hasan I, Rashid T, Jaikaransingh V, Heilig C, Abdel-Rahman EM, Awad AS (2024). SGLT2 inhibitors: Beyond glycemic control. J Clin Transl Endocrinol.

[42] Perry RJ, Shulman GI (2020). Sodium-glucose cotransporter-2 inhibitors: Understanding the mechanisms for therapeutic promise and persisting risks. J Biol Chem.

[43] Seidu S, Alabraba V, Davies S, Newland-Jones P, Fernando K, Bain SC (2024). SGLT2 Inhibitors - The New Standard of Care for Cardiovascular, Renal and Metabolic Protection in Type 2 Diabetes: A Narrative Review. Diabetes Ther.

[44] Verma S, McMurray JJV (2018). SGLT2 inhibitors and mechanisms of cardiovascular benefit: a state-of-the-art review. Diabetologia.

[45] Xu B, Li S, Kang B, Zhou J (2022). The current role of sodium-glucose cotransporter 2 inhibitors in type 2 diabetes mellitus management. Cardiovasc Diabetol.

[46] Wang D, Naumova A, Isquith D, Sapp J, Huynh KA, Tucker I (2024). Dapagliflozin Reduces Systemic Inflammation in Patients with Type 2 Diabetes Without Known Heart Failure. Cardiovasc Diabetol.

[47] Wanner C, Lachin JM, Inzucchi SE, Fitchett D, Mattheus M, George J (2018). Empagliflozin and Clinical Outcomes in Patients With Type 2 Diabetes Mellitus, Established Cardiovascular Disease, and Chronic Kidney Disease. Circulation.

[48] Thomson SC, Rieg T, Miracle C, Mansoury H, Whaley J, Vallon V (2012). Acute and chronic effects of SGLT2 blockade on glomerular and tubular function in the early diabetic rat. Am J Physiol Regul Integr Comp Physiol.

[49] Cherney DZI, Dekkers CCJ, Barbour SJ, Cattran D, Abdul Gafor AH, Greasley PJ (2020). Effects of the SGLT2 inhibitor dapagliflozin on proteinuria in non-diabetic patients with chronic kidney disease (DIAMOND): a randomised, double-blind, crossover trial. Lancet Diabetes Endocrinol.

[50] Vallon V, Verma S (2021). Effects of SGLT2 Inhibitors on Kidney and Cardiovascular Function. Annu Rev Physiol.

[51] Matsusaka Y, Chen X, Arias-Loza P, Werner RA, Nose N, Sasaki T (2022). In Vivo Functional Assessment of Sodium-Glucose Cotransporters (SGLTs) Using [(18)F]Me4FDG PET in Rats. Mol Imaging.

[52] Chen X, Werner RA, Koshino K, Nose N, Muhlig S, Rowe SP (2022). Molecular Imaging-Derived Biomarker of Cardiac Nerve Integrity - Introducing High NET Affinity PET Probe (18)F-AF78. Theranostics.

[53] Nishimura M, Tsukamoto K, Hasebe N, Tamaki N, Kikuchi K, Ono T (2008). Prediction of cardiac death in hemodialysis patients by myocardial fatty acid imaging. J Am Coll Cardiol.

[54] DeGrado TR, Bhattacharyya F, Pandey MK, Belanger AP, Wang S (2010). Synthesis and preliminary evaluation of 18-(18)F-fluoro-4-thia-oleate as a PET probe of fatty acid oxidation. J Nucl Med.

[55] Sartor O, de Bono J, Chi KN, Fizazi K, Herrmann K, Rahbar K (2021). Lutetium-177-PSMA-617 for Metastatic Castration-Resistant Prostate Cancer. N Engl J Med.

[56] Toyama Y, Werner RA, Ruiz-Bedoya CA, Ordonez AA, Takase K, Lapa C (2021). Current and future perspectives on functional molecular imaging in nephro-urology: theranostics on the horizon. Theranostics.

[57] Wieland DM, Wu J, Brown LE, Mangner TJ, Swanson DP, Beierwaltes WH (1980). Radiolabeled adrenergi neuron-blocking agents: adrenomedullary imaging with [131I]iodobenzylguanidine. J Nucl Med.

[58] Lindner T, Loktev A, Altmann A, Giesel F, Kratochwil C, Debus J (2018). Development of Quinoline-Based Theranostic Ligands for the Targeting of Fibroblast Activation Protein. J Nucl Med.

[59] Singh S, Garg A, Tantry US, Bliden K, Gurbel PA, Gulati M (2024). Cardiovascular Outcomes With Empagliflozin and Dapagliflozin in Patients Without Diabetes. Am J Cardiol.

